# Manual Tibial Distraction in Seated Position for Identification of Intra-articular Knee Joint Lesions: A Case Report Presenting a Novel Clinical Special Test

**DOI:** 10.7759/cureus.77837

**Published:** 2025-01-22

**Authors:** Aaron P Tillman, Cory J Dixon, Sawyer Longley, Britton A Ethridge, Julieanne P Sees

**Affiliations:** 1 Department of Research, Alabama College of Osteopathic Medicine, Dothan, USA; 2 Haub School of Business, St. Joseph's University/American Osteopathic Association, Chicago, USA

**Keywords:** distraction test, intra-articular tear, knee pain, meniscus, osteoarthritis, osteopathy, special test

## Abstract

The meniscus is a weight-bearing, intra-articular, fibrocartilaginous structure that is frequently injured. Traditional special tests for diagnosis of intra-articular lesions of the knee involve provocative tests with compressive or torsional components, often leading to increased pain. With this case study, we aim to introduce the Tibial Distraction Test (TDT), a novel clinical special test for the assessment of intra-articular knee joint lesions, including meniscus injury and osteoarthritis (OA), without provoking pain.

A 30-year-old man presented to the clinic with right knee pain and swelling of two months duration following a forceful extension and internal rotation motion which led to a catch then audible pop and discomfort. The patient’s initial assessment included McMurray's test, joint line palpation, and Apley’s compression tests with positive results for each test. A second examiner, blinded to the initial examination, assessed the patient using the proposed TDT. The patient reported pain relief, indicating a positive result. Subsequent magnetic resonance imaging (MRI) confirmed the diagnosis of a medial meniscus tear. Arthroscopy showed a full-thickness meniscal tear and debridement was performed. At one month's follow-up, the patient returned to baseline function with resolution of symptoms.

Diagnosing intra-articular lesions of the knee requires a thorough history and examination, frequently including painful provocative clinical special tests in a patient already presenting with pain as a primary complaint. Our case presents a novel manual clinical special test in a seated position which appears to be a pain-relieving test that may be used to assess for intra-articular knee joint lesions.

## Introduction

The menisci are fibrocartilaginous plates on the proximal articular surface of the tibia. They act to deepen the knee joint and provide shock absorption during weight-bearing activities [1]. Meniscal injuries affect 0.61-0.70 per 1000 people in the United States each year [2]. According to Majewski et al., meniscus injuries account for 15.5% of knee injuries in athletes alone [3]. Diagnosing meniscus tears can involve patient history, thorough inspection, physical examination, special tests, and imaging. Special tests including McMurray’s, Thessaly’s, Apley’s, and Joint Line Tenderness require the provocation of pain, tenderness, catching, or locking sensations for indication of possible meniscus injury and have been shown to have limited clinical diagnostic accuracy [4]. The benchmark diagnostic tool, however, is magnetic resonance imaging (MRI) alongside confirmatory arthroscopy [5]. 

Knee osteoarthritis (OA) is a leading cause of disability and the knee joint is the most common location of all osteoarthropathy. It is the result of degenerative changes to the meniscus and alterations to the structure of joint surface collagen and proteoglycans leading to subchondral bone sclerosis and synovial membrane changes. Recent data suggests knee OA prevalence between 9.6% and 19%, with a higher prevalence with advancing age. In the US, one study found a prevalence of 16%, while another showed an 8.2% difference in prevalence between women and men, 19.1% and 10.9% respectively [6,7]. Since OA is usually a clinical diagnosis, the physical exam and history are vital in making a diagnosis. Clinicians use clues such as symptomatology, X-ray findings, laboratory values, physical signs, and other imaging to help determine OA in a patient [8]. 

Manual, mechanical, and surgical knee joint distraction (KJD) have been used successfully as treatment approaches for knee osteoarthritis with improvements in knee pain and function following treatment [9-11]. Surgical unloading of the knee via KJD may slow the progress of OA by allowing the cartilage to promote bone healing and adapt to the loss of articular cartilage due to joint loading [9,11]. 

Current bedside testing for intra-articular lesions such as meniscal tears and knee OA correlate positive findings with provocative pain upon examination. The Tibial Distraction Test (TDT) however aims to minimize additional pain provoked during standard clinical examination for the assessment of intra-articular knee joint lesions. To further assess this, the case of a 30-year-old man presenting with knee pain and intermittent swelling is described to introduce this novel prospective clinical special test using manual tibial distraction in a seated position for the identification of intra-articular knee joint lesions. It is hypothesized that the distraction force applied to the knee with the TDT decreases compression on joint surface structures, including the meniscus and articular cartilage, decreasing patient pain perception.

## Case presentation

**Figure 1:**
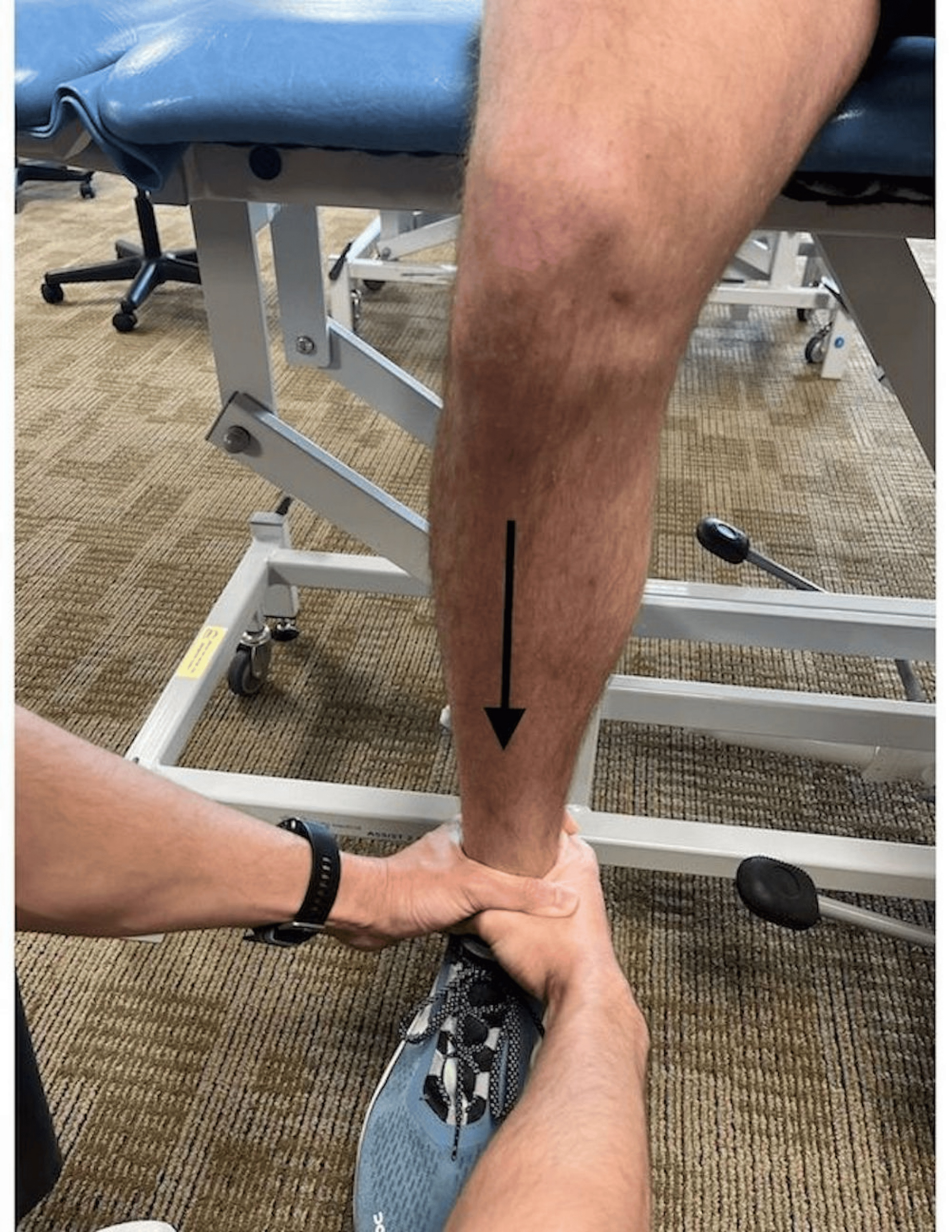
Tibial Distraction Test. With the patient in a seated position, thigh supported by a table, and lower leg hanging from the table, the examiner applies an inferior traction force through the tibia - gapping the tibiofemoral joint. Reduction in or alleviation of pain symptoms indicates a positive result, suggesting an intra-articular cause of symptoms.

A 30-year-old man presented with right knee pain of approximately two months duration. The initial injury occurred after a forceful extension and internal rotation motion triggered a catch then an audible pop, followed by knee pain and swelling. Over the next two months, the patient experienced variable levels of knee pain and swelling following physical activity. The patient was examined by an initial examiner using McMurray's, joint line palpation, and Apley’s compression tests with positive results for each test, suggesting meniscal injury. A second examiner, blinded to the results of the first, examined the patient using the proposed TDT (Figure 1). The patient reported pain relief during the test, indicating a positive result and likely intra-articular tissue involvement.

**Figure 2:**
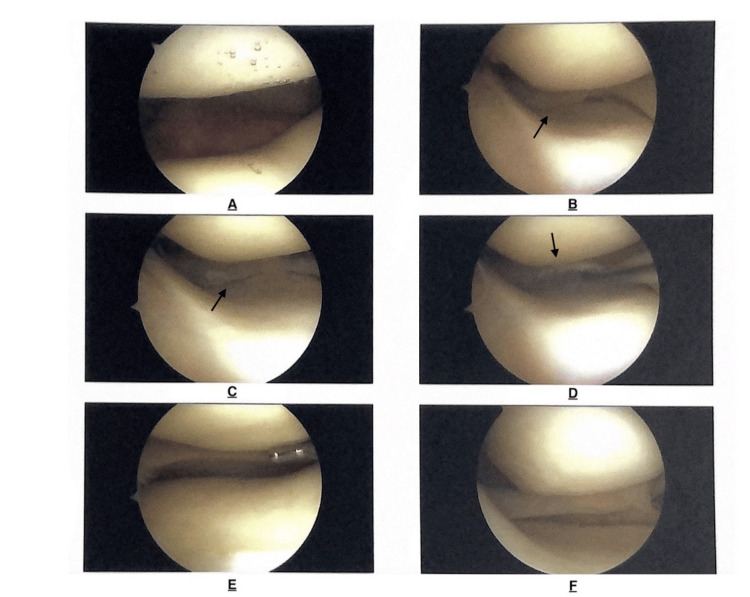
Intraoperative knee arthroscopy images. A: patellofemoral joint surfaces without lesions. B-D: Medial tibiofemoral compartment with presence of medial meniscus tear. (black arrows) E: Lateral tibiofemoral compartment without meniscus lesion. F: Medial compartment following debridement of medial meniscus

Follow-up MRI was positive for medial meniscus tear indicating arthroscopic debridement. During surgery, a full-thickness meniscal tear was identified and debrided (Figure 2). Following surgical debridement, the patient underwent four weeks of physical therapy and returned to baseline function with full resolution of knee pain.

## Discussion

Diagnosing meniscal injury involves evaluation of the patient history, thorough musculoskeletal inspection, physical examination, special tests, and imaging. The benchmark for diagnosing meniscus lesions (non-surgically) is MRI. MRI has been used alongside knee arthroscopy for the diagnosis and treatment of meniscus lesions [6]. Historically, special tests, also known as physical diagnosing tests, have been an integral part of diagnosing meniscus lesions. There are many established special tests for meniscal injuries such as Apley’s Compression test, McMurray’s test, Thessaly’s test, Bounce Home test, Steinman’s tenderness displacement test, and joint line palpation [4,12]. For each of these tests, a reproduction of pain and/or palpable catch or click indicates a positive result, but the eliciting pain may not be a viable option in some patient populations. 

A multidisciplinary consensus study found that knee OA can be clinically diagnosed by the presence of symptoms of persistent knee pain, stiffness in the morning, and limited function, combined with the signs of joint crepitus, restricted movement and bony enlargement [13]. Radiographic imaging can be used for diagnosis and progression of OA using plain films in two planes and may include findings of osteophyte formation, joint space narrowing and obliteration, subchondral sclerosis and cyst formation [14]. Abhishek and Doherty argue that OA may be an asymptomatic finding on imaging and so even if there is a diagnosis of OA on imaging, the patient may not be experiencing symptoms of OA. Thus the medical provider must treat the patient not the radiologic scan [15].

Traditional special tests involve provocative compressive or torsional components often increasing pain in patients. Utilizing a bedside test without reproduction of pain is especially useful if a patient has limited mobility, high pain reactivity, or the clinician’s goal is to minimize patient positional changes during examination. The case exemplifies that the TDT has similar clinical utility as current bedside testing for meniscal tears, while also keeping patients free of additional pain. 

Distraction of the tibia gaps the tibiofemoral joint, reducing compressive forces on the menisci and tibiofemoral joint surfaces [10]. Relief of symptoms with tibial distraction should then, theoretically, indicate an intra-articular etiology such as meniscus injury or OA. An increase in pain with tibial distraction would, conversely, indicate an extra-articular etiology, similar to that of Apley’s distraction test. For example, Apley’s special test elicits knee pain during compression in patients with intra-articular lesions and knee pain with distraction in patients with extra-articular lesions [12]. Therefore, theoretically, the results of the TDT would then help to guide further examination of either intra or extra-articular etiologies.

A study performed by Choi and Lee in 2019 compared two groups who underwent knee joint traction therapy and general physical therapy for degenerative arthritis. After four weeks of treatment, they found decreased pain and increased function in both groups but found a more significant change in those who had received knee joint traction therapy [10]. This demonstrates the effectiveness of tibial distraction in decreasing intra-articular pain effectively. 

Additionally, tibiofemoral joint distraction is used in the rehabilitative setting for the relief of intra-articular knee pain often associated with OA [10]. Surgically, KJD has been utilized to reduce the symptoms associated with and even improve OA of the knee [9,10]. It would be reasonable then to presume that manual tibial distraction should alleviate joint pain due to intra-articular lesions such as meniscus tears and OA in the function of a clinical special test.

This test is, however, limited in its immediate applicability to the general population due to the single-subject nature of this case report. The authors recommend further study, including but not limited to case series or randomized control trials to assess the accuracy and validity of the TDT and applicability across diverse patient populations.

## Conclusions

Current standard special tests assessing for possible intra-articular knee lesions rely on eliciting pain during compression to indicate a positive result. This case study demonstrates a novel manual clinical special test where a positive result is indicated by pain relief, thus providing an improved patient experience while also providing a reliable indication of intra-articular knee joint lesions. Further research is needed to evaluate the accuracy, validity and applicability of the TDT prior to routine application in the clinical setting.
